# Surgical Decompression of Trigger Finger

**Published:** 2014-09-01

**Authors:** Karan Chopra, Garth N. Walker, Kashyap K. Tadisina, Scott D. Lifchez

**Affiliations:** ^a^Department of Plastic and Reconstructive Surgery, Johns Hopkins School of Medicine, Baltimore, Md; ^b^Division of Plastic Surgery, University of Maryland School of Medicine, Baltimore, Md; ^c^College of Medicine, University of Illinois, Chicago, Ill

**Keywords:** stenosing tenosynovitis, trigger finger, surgical release, A1 pulley, hand surgery

**Figure:**
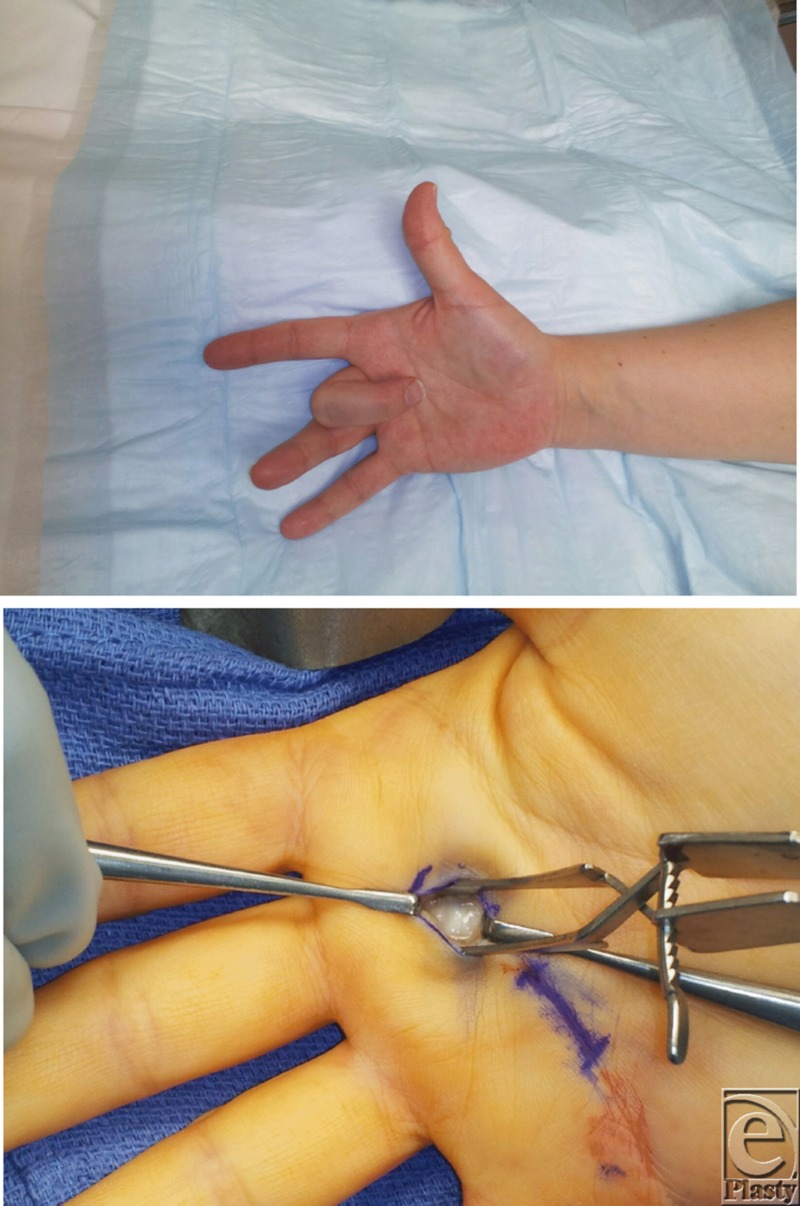


**Figure:**
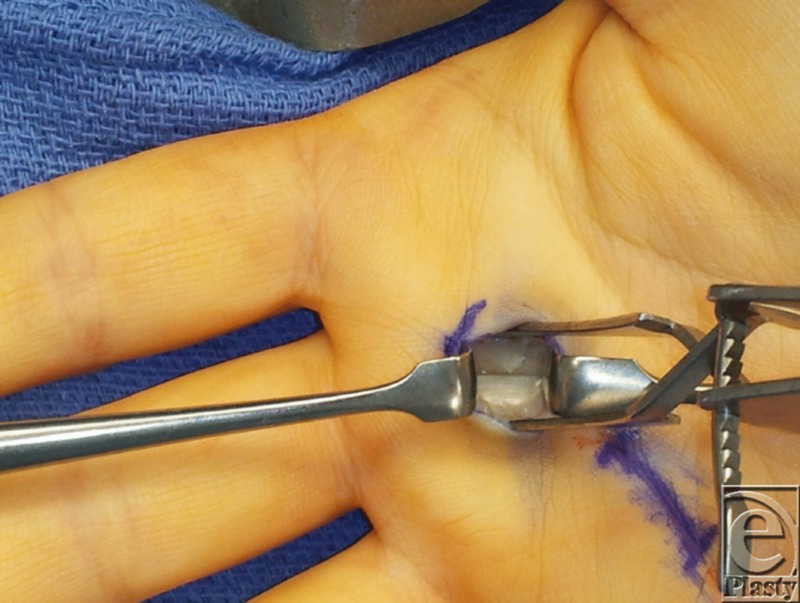


## DESCRIPTION

A 41-year-old right-hand-dominant woman presented with a longstanding history of right middle finger trigger digit that responded to corticosteroid injections but continued to recur. She presents for definitive treatment of her condition.

## QUESTIONS

1. **What is the etiology of trigger finger and how does it present?**
2. **What are the nonsurgical treatments for trigger finger and when is surgical release indicated?**
3. **What are the basic principles of surgical release?**
4. **What anatomic structures need to be identified and protected during surgical release?**

## DISCUSSION

The pathology of stenosing tenosynovitis, also known as trigger finger, lies in the mismatch between the size of the flexor tendon sheath and its contents within the first annular (A-1 pulley). Repeated attempts of the tendon to glide through the stenotic sheath ultimately leads to inflammation and the inability to flex or extend the digit smoothly. This initially starts as a clicking that may be painless in nature and may eventually progress to a finger that becomes locked in flexion. This often requires passive manipulation of the pathologic finger into extension. These patients often present with pain localization to the palm, metacarpophalangeal (MCP) or proximal interphalageal joint (PIP).^1^

Nonsurgical modalities include steroid injection and splinting.^2^^,^^3^ At our center, nonsurgical treatment generally involves local steroid injection assuming the patient has no active osteoarthritis or signs of active infection.^1^^,^^3^ If symptoms persist after this period, surgical intervention is strongly considered.

The prime focus during operative management lies in surgical release of the entrapped tendon. A transverse incision is made on the volar aspect of the hand overlying the MCP joint and the A1 pulley. Dissection is carried out down to the A1 pulley. Special attention must be paid to the radial and ulnar sides of the pathologic digit to avoid neurovascular injury.^1^ The pulley is then divided, freeing the tendon beneath it. Ultimately, the goal of the procedure is complete release of the pulley of the pathologic digit with preservation of all other pulleys and structures.

Although complications are rare for open surgical approaches, proper understanding of the relevant anatomy, and landmarks should be taken into consideration. Landmarks to be cognizant of are the PIP to the palmar digital crease (PDC) distance, to identify the proximal edge of the A1 pulley. On average the PIP to PDC distance is 2.42 cm for index, long, and ring fingers, so any abnormal variance from this measure should require finer dissection for any gross abnormality. The index finger and small fingers pose a great challenge to release particularly because of the oblique route of the flexor tendon sheath courses, and percutaneous approach poses great risk prior to release.^1^ For the thumb, the ulnar side should be evaluated with care due to the oblique ulnar course of the radial digital artery.

## References

[1] Ryzewicz M, Wolf JM (2006). Trigger digits: principles, management, and complications. J Hand Surg Am.

[2] Hazani R, Engineer NJ, Zeineh LL, Wilhelmi BJ (2008). Assessment of the distal extent of the A1 pulley release: a new technique. Eplasty.

[3] Wang J, Zhao JG, Liang CC (2013). Percutaneous release, open surgery, or corticosteroid injection, which is the best treatment method for trigger digits?. Clin Orthop Relat Res.

[4] Fowler JR, Baratz ME (2013). Percutaneous trigger finger release. J Hand Surg Am.

[5] Lee WT, Chong AK (2011). Outcome study of open trigger digit release. J Hand Surg Eur.

[6] Sato ES, Gomes Dos Santos JB, Belloti JC, Albertoni WM, Faloppa F (2012). Treatment of trigger finger: randomized clinical trial comparing the methods of corticosteroid injection, percutaneous release and open surgery. Rheumatology (Oxford).

